# Supplementary material for: Presynaptic inhibition of dopamine neurons controls optimistic bias
Source: eLife. 2021 Jun 1;10:e64907. doi: 10.7554/eLife.64907 (PMC8169112; doi:10.7554/eLife.64907)
Supplement: Supplementary file 1. [file elife-64907-supp1.docx]

Table S1. List of fly strains and crosses for experiments, and the statistical results

| Figure | Group code | Males | Females | *N* | *D'Agostino (Shapiro-Wilk) normality test*  *(P value)* | *Brown-Forsythe test (P value)* | *Statistical test for group comparisons*  *(Adjusted P value)* |
| --- | --- | --- | --- | --- | --- | --- | --- |
| Figure 1A | *1* | *CS* | *CS* | *23* | *0.6598* | *NA* | *0.7721 (1 vs. 2)*  *0.43404 (1 vs. 3)*  *> 0.9999 (1 vs. 4)*  *0.3042 (1 vs. 5)*  *0.9798 (1 vs. 6)*  *0.0162 (1 vs. 7) **  *Mann-Whitney test &*  *FDR (Benjamini & Hochberg)* |
|  | *2* | *yv;UAS-Grd-RNAi* | *w;;R58E02-GAL4* | *8* | *0.778* |  |  |
|  | *3* | *yv;UAS-Lcch3-RNAi* | *w;;R58E02-GAL4* | *8* | *0.8472* |  |  |
|  | *4* | *yv;UAS-Rdl-RNAi* | *w;;R58E02-GAL4* | *8* | *0.5397* |  |  |
|  | *5* | *yv;UAS-GABA-B-R1-RNAi* | *w;;R58E02-GAL4* | *8* | *0.4446* |  |  |
|  | *6* | *yv;UAS-GABA-B-R2-RNAi* | *w;;R58E02-GAL4* | *8* | *0.0158 ** |  |  |
|  | *7* | *yv;UAS-GABA-B-R3-RNAi* | *w;;R58E02-GAL4* | *8* | *0.2946* |  |  |
| Figure 1B | *1* | *CS* | *w;Tub-GAL80ts;R58E02-GAL4* | *12* | *0.2044* | *0.5544*  *NA* | *0.0062 (1 vs. 2) ***  *0.0123 (2 vs. 3) **  *Sidak’s test*  *> 0.9999 (4 vs. 5)*  *> 0.9999 (5 vs. 6)*  *Dunn’s test* |
|  | *2* | *yv;UAS-GABA-B-R3-RNAi* | *w;Tub-GAL80ts;R58E02-GAL4* | *12* | *0.5883* |  |  |
|  | *3* | *yv;UAS-GABA-B-R3-RNAi* | *CS* | *10* | *0.6638* |  |  |
|  | *4* | *CS* | *w;Tub-GAL80ts;R58E02-GAL4* | *8* | *0.6718* |  |  |
|  | *5* | *yv;UAS-GABA-B-R3-RNAi* | *w;Tub-GAL80ts;R58E02-GAL4* | *7* | *NA* |  |  |
|  | *6* | *yv;UAS-GABA-B-R3-RNAi* | *CS* | *8* | *0.5004* |  |  |
| Figure 1C,D | *1* | *yw;GABA-B-R3-T2A-GAL4* | *w;;UAS-mCD8::GFP* | *NA* | *NA* | *NA* | *NA* |
| Figure 1E | *1* | *CS* | *w;;R58E02-GAL4* | *12x4* | *NA* | *NA* | *Asymptote*  *0.023 (1 vs. 2) **  *0.049 (2 vs. 3 )**  *Speed*  *0.656 (1 vs. 2)*  *0.441 (2 vs. 3)*  *Hyperbola curve fitting & permutation test (cf. Fig. S1E,F)* |
|  | *2* | *yv;UAS-GABA-B-R3-RNAi* | *w;;R58E02-GAL4* | *12x4* | *NA* |  |  |
|  | *3* | *yv;UAS-GABA-B-R3-RNAi* | *w* | *12x4* | *NA* |  |  |
| Figure 1H | *1* | *w;UAS-GCaMP6s* | *w;;R15A04-GAL4* | *7* | *0.4453/0.6459* | *0.1131* | *0.0491 (1 vs. 2, γ5) **  *0.0491 (1 vs. 2, α1) **  *Holm-Sidak's test* |
|  | *2* | *w;UAS-GCaMP6s* | *w;UAS-GABA-B-R3-RNAi;R15A04-GAL4* | *8* | *0.3074/0.3765* |  |  |
| Figure 2B | *1* | *w;;GABA-B-R3-sfGFP-TVPTBF* | *w;UAS-mCD8::RFP; R58E02-GAL4* | *NA* | *NA* | *NA* | *NA* |
| Figure 2C,D | *1* | *w;R58E02-LexA; VT043924-GAL4* | *w;UAS-mCD8::RFP; LexAop-rCD2::GFP* | *NA* | *NA* | *NA* | *NA* |
| Figure 2E | *1* | *w* | *w;;UAS-VGAT-RNAi* | *11* | *0.8519* | *0.7020* | *0.0008 (1 vs. 2) ****  *0.0023 (1 vs. 4) ***  *0.0004 (2 vs. 3) ****  *0.0171 (4 vs. 5) **  *Sidak’s test* |
|  | *2* | *w;;VT043924-GAL4* | *w;;UAS-VGAT-RNAi* | *12* | *0.4823* |  |  |
|  | *3* | *w* | *w;UAS-Gad1-RNAi* | *13* | *0.7426* |  |  |
|  | *4* | *w;;VT043924-GAL4* | *w;UAS-Gad1-RNAi* | *12* | *0.0929* |  |  |
|  | *5* | *w;;VT043924-GAL4* | *w* | *11* | *0.4354* |  |  |
| Figure 2H | *1* | *w;UAS-GCaMP6s* | *w;;R15A04-GAL4* | *6* | *NA* | *NA* | *0.0455 (1 vs. 2 terminal) **  *> 0.9999 (1 vs. 2 dendrite)*  *Dunn’s test* |
|  | *2* | *w;UAS-GCaMP6s* | *w;UAS-GABA-B-R3-RNAi;R15A04-GAL4* | *7* | *NA* |  |  |
| Figure 3A | *1* | *UAS-GCaMP6s. brpS.mCherry* | *w;;R15A04-GAL4* | *NA* | *NA* | *NA* | *NA* |
| Figure 3C | *1* | *UAS-GCaMP6s. brpS.mCherry* | *w;;R15A04-GAL4* | *24* | *< 0.0001 ** | *NA* | *0.0004 (1 vs. 2) ****  *Mann-Whitney test* |
|  | *2* | *UAS-GCaMP6s. brpS.mCherry* | *w;UAS-GABA-B-R3-RNAi;R15A04-GAL4* | *17* | *0.2523* |  |  |
| Figure 3E | *1* | *UAS-GCaMP6s. brpS.mCherry* | *w;;R15A04-GAL4* | *16* | *NA* | *NA* | *NA* |
|  | *2* | *UAS-GCaMP6s. brpS.mCherry* | *w;UAS-GABA-B-R3-RNAi;R15A04-GAL4* | *11* | *NA* |  |  |
| Figure 3G | *1* | *UAS-GCaMP6s. brpS.mCherry* | *w;;R15A04-GAL4* | *22* | *0.0083 **** | *NA*  *0.7022* | *0.0182 (1 vs. 2) **  *Mann-Whitney test*  *0.024(3 vs. 4) **  *Unpaired t-test* |
|  | *2* | *UAS-GCaMP6s. brpS.mCherry* | *w;UAS-GABA-B-R3-RNAi;R15A04-GAL4* | *17* | *0.9976* |  |  |
|  | *3* | *UAS-GCaMP6s. brpS.mCherry* | *w;;R15A04-GAL4* | *15* | *0.567* |  |  |
|  | *4* | *UAS-GCaMP6s. brpS.mCherry* | *w;UAS-GABA-B-R3-RNAi;R15A04-GAL4* | *11* | *0.8439* |  |  |
| Figure 4A | *1 (100:0)* | *CS* | *w;;R58E02-GAL4* | *14* | *0.7168* | *0.2562* | *0.0678 (1 vs. 2)*  *0.0036 (2 vs. 3) ***  *Sidak’s test* |
|  | *2 (100:0)* | *yv;UAS-GABA-B-R3-RNAi* | *w;;R58E02-GAL4* | *17* | *0.1584* |  |  |
|  | *3 (100:0)* | *yv;UAS-GABA-B-R3-RNAi* | *w* | *16* | *0.4368* |  |  |
| Figure 4A | *1 (50:50)* | *CS* | *w;;R58E02-GAL4* | *10* | *0.1595* | *0.6282* | *0.0006 (1 vs. 2) ****  *0.0001 (2 vs. 3) ****  *Sidak’s test* |
|  | *2 (50:50)* | *yv;UAS-GABA-B-R3-RNAi* | *w;;R58E02-GAL4* | *11* | *0.5046* |  |  |
|  | *3 (50:50)* | *yv;UAS-GABA-B-R3-RNAi* | *w* | *11* | *0.5064* |  |  |
| Figure 4A | *1 (0:100)* | *CS* | *w;;R58E02-GAL4* | *8* | *0.7315* | *0.1939* | *0.0705 (1 vs. 2)*  *0.5314 (2 vs. 3)*  *Sidak’s test* |
|  | *2 (0:100)* | *yv;UAS-GABA-B-R3-RNAi* | *w;;R58E02-GAL4* | *8* | *0.842* |  |  |
|  | *3 (0:100)* | *yv;UAS-GABA-B-R3-RNAi* | *w* | *8* | *0.6902* |  |  |

| Figure 4B | *1 (100:0)* | *CS* | *w;;R58E02-GAL4* | *20* | *0.6823* | *0.8934* | *0.0108 (1 vs. 2) **  *0.0138 (2 vs. 3) **  *Sidak’s test* |
| --- | --- | --- | --- | --- | --- | --- | --- |
|  | *2 (100:0)* | *yv;UAS-GABA-B-R3-RNAi* | *w;;R58E02-GAL4* | *20* | *0.9439* |  |  |
|  | *3 (100:0)* | *yv;UAS-GABA-B-R3-RNAi* | *w* | *20* | *0.4934* |  |  |
| Figure 4B | *1 (50:50)* | *CS* | *w;;R58E02-GAL4* | *17* | *0.6754* | *0.3323* | *0.0028 (1 vs. 2) ***  *0.0073 (2 vs. 3) ***  *Sidak’s test* |
|  | *2 (50:50)* | *yv;UAS-GABA-B-R3-RNAi* | *w;;R58E02-GAL4* | *17* | *0.2405* |  |  |
|  | *3 (50:50)* | *yv;UAS-GABA-B-R3-RNAi* | *w* | *14* | *0.2775* |  |  |
| Figure 4B | *1 (0:100)* | *CS* | *w;;R58E02-GAL4* | *8* | *0.9817* | *0.1037* | *0.2464 (1 vs. 2)*  *0.0611 (2 vs. 3)*  *Sidak’s test* |
|  | *2 (0:100)* | *yv;UAS-GABA-B-R3-RNAi* | *w;;R58E02-GAL4* | *8* | *0.6864* |  |  |
|  | *3 (0:100)* | *yv;UAS-GABA-B-R3-RNAi* | *w* | *8* | *0.7464* |  |  |
| Figure 4C | *1 (100:0)* | *w;;VT043924-GAL4* | *w* | *12* | *0.0898* | *0.1464* | *0.156 (1 vs. 2)*  *0.0328 (2 vs. 3) **  *Sidak’s test* |
|  | *2 (100:0)* | *w;;VT043924-GAL4* | *w;UAS-Gad1-RNAi* | *12* | *0.721* |  |  |
|  | *3 (100:0)* | *w* | *w;UAS-Gad1-RNAi* | *12* | *0.7745* |  |  |
| Figure 4C | *1 (50:50)* | *w;;VT043924-GAL4* | *w* | *11* | *0.8654* | *0.7088* | *0.002 (1 vs. 2) ***  *0.0002 (2 vs. 3) ****  *Sidak’s test* |
|  | *2 (50:50)* | *w;;VT043924-GAL4* | *w;UAS-Gad1-RNAi* | *10* | *0.2975* |  |  |
|  | *3 (50:50)* | *w* | *w;UAS-Gad1-RNAi* | *11* | *0.7011* |  |  |
| Figure 4C | *1 (0:100)* | *w;;VT043924-GAL4* | *w* | *9* | *0.2449* | *NA* | *0.1923 (1 vs. 2)*  *0.2441 (2 vs. 3)*  *Dunn’s test* |
|  | *2 (0:100)* | *w;;VT043924-GAL4* | *w;UAS-Gad1-RNAi* | *10* | *0.0136 ** |  |  |
|  | *3 (0:100)* | *w* | *w;UAS-Gad1-RNAi* | *9* | *0.4281* |  |  |
| Figure 4D | *1 (100:0)* | *w;;VT043924-GAL4* | *w* | *12* | *0.8619* | *0.122* | *0.0141 (1 vs. 2) **  *0.0016 (2 vs. 3) ***  *Sidak’s test* |
|  | *2 (100:0)* | *w;;VT043924-GAL4* | *w;UAS-Gad1-RNAi* | *12* | *0.7042* |  |  |
|  | *3 (100:0)* | *w* | *w;UAS-Gad1-RNAi* | *12* | *0.0772* |  |  |
| Figure 4D | *1 (50:50)* | *w;;VT043924-GAL4* | *w* | *12* | *0.144* | *0.334* | *0.0323 (1 vs. 2) **  *0.0167 (2 vs. 3) **  *Sidak’s test* |
|  | *2 (50:50)* | *w;;VT043924-GAL4* | *w;UAS-Gad1-RNAi* | *11* | *0.6469* |  |  |
|  | *3 (50:50)* | *w* | *w;UAS-Gad1-RNAi* | *12* | *0.3738* |  |  |
| Figure 4D | *1 (0:100)* | *w;;VT043924-GAL4* | *w* | *12* | *0.5633* | *0.2038* | *0.6469 (1 vs. 2)*  *0.3052 (2 vs. 3)*  *Sidak’s test* |
|  | *2 (0:100)* | *w;;VT043924-GAL4* | *w;UAS-Gad1-RNAi* | *12* | *0.7223* |  |  |
|  | *3 (0:100)* | *w* | *w;UAS-Gad1-RNAi* | *12* | *0.2042* |  |  |

| Figure 1-figure supplement1A | *1* | *CS* | *w;;R58E02-GAL4* | *12* | *0.3276* | *0.8355* | *0.0213 (1 vs. 2) **  *0.0195 (2 vs. 3) **  *Sidak’s test* |
| --- | --- | --- | --- | --- | --- | --- | --- |
|  | *2* | *yv;UAS-GABA-B-R3-RNAi* | *w;;R58E02-GAL4* | *12* | *0.4965* |  |  |
|  | *3* | *yv;UAS-GABA-B-R3-RNAi* | *w* | *12* | *0.2284* |  |  |
| Figure 1-figure supplement1AB | *1* | *CS* | *w;;R58E02-GAL4, UAS-Dicer2* | *12* | *0.6802* | *0.8719* | *0.0059 (1 vs. 2) ***  *0.004 (2 vs. 3) ***  *Sidak’s test* |
|  | *2* | *yv;UAS-GABA-B-R3-RNAi #2* | *w;;R58E02-GAL4, UAS-Dicer2* | *11* | *0.8289* |  |  |
|  | *3* | *yv;UAS-GABA-B-R3-RNAi #2* | *w* | *12* | *0.6459* |  |  |
| Figure 1-figure supplement1D | *1(5s)* | *CS* | *CS* | *8* | *0.6381* | *0.9178* | *0.5505 (1 vs. 2)*  *< 0.0001 (1 vs. 3) ****  *< 0.0001 (1 vs. 4) ****  *< 0.0001 (1 vs. 5) ****  *0.0009 (2 vs. 3) ****  *< 0.0001 (2 vs. 4) ****  *< 0.0001 (2 vs. 5) ****  *0.0109 (3 vs. 4) **  *0.0016 (3 vs. 5) ***  *0.972 (4 vs. 5)*  *Tukey's test* |
|  | *2(10s)* | *CS* | *CS* | *8* | *0.7601* |  |  |
|  | *3(20s)* | *CS* | *CS* | *16* | *0.0663* |  |  |
|  | *4(60s)* | *CS* | *CS* | *16* | *0.6194* |  |  |
|  | *5(120s)* | *CS* | *CS* | *16* | *0.6152* |  |  |
| Figure 2-figure supplement1A | *1* | *w;;GABA-B-R3-sfGFP-TVPTBF* | *w;UAS-mCD8::RFP; R58E02-GAL4* | *NA* | *NA* | *NA* | *NA* |
| Figure 2-figure supplement1B | *1* | *w;;R15A04-GAL4* | *w;UAS-mCD8::GFP* | *NA* | *NA* | *NA* | *NA* |
| Figure 2-figure supplement1C | *1* | *w;UAS-GCaMP6s, UAS-mCD8::RFP* | *w;;R15A04-GAL4* | *NA* | *NA* | *NA* | *NA* |
| Figure 3-figure supplement1B | *1* | *UAS-GCaMP6s. brpS.mCherry* | *w;;R15A04-GAL4* | *NA* | *NA* | *NA* | *NA* |
|  | *2* | *UAS-GCaMP6s. brpS.mCherry* | *w;UAS-GABA-B-R3-RNAi;R15A04-GAL4* |  |  |  |  |
| Figure 3-figure supplement1C | *1* | *UAS-GCaMP6s. brpS.mCherry* | *w;;R15A04-GAL4* | *8* | *NA* | *NA* | *NA* |
|  | *2* | *UAS-GCaMP6s. brpS.mCherry* | *w;UAS-GABA-B-R3-RNAi;R15A04-GAL4* | *6* |  |  |  |
| Figure 3-figure supplement1D | *1* | *UAS-GCaMP6s. brpS.mCherry* | *w;;R15A04-GAL4* | *16* | *0.2152* | *0.0001 **** | *0.0501 (1 vs. 2)*  *Mann-Whitney test* |
|  | *2* | *UAS-GCaMP6s. brpS.mCherry* | *w;UAS-GABA-B-R3-RNAi;R15A04-GAL4* | *11* | *0.9832* |  |  |
| Figure 3-figure supplement1E | *1* | *UAS-GCaMP6s. brpS.mCherry* | *w;;R15A04-GAL4* | *8* | *NA* | *NA* | *NA* |
|  | *2* | *UAS-GCaMP6s. brpS.mCherry* | *w;UAS-GABA-B-R3-RNAi;R15A04-GAL4* | *6* |  |  |  |
| Figure 3-figure supplement1F | *1* | *UAS-GCaMP6s. brpS.mCherry* | *w;;R15A04-GAL4* | *22* | *0.3783* | *0.459* | *0.0271 (1 vs. 2) **  *Unpaired t-test* |
|  | *2* | *UAS-GCaMP6s. brpS.mCherry* | *w;UAS-GABA-B-R3-RNAi;R15A04-GAL4* | *17* | *0.5638* |  |  |
